# Bioactive Cembranoids from the South China Sea Soft Coral *Sarcophyton elegans*

**DOI:** 10.3390/molecules200713324

**Published:** 2015-07-22

**Authors:** Xin Liu, Junsheng Zhang, Qiao Liu, Guihua Tang, Hongsheng Wang, Chengqi Fan, Sheng Yin, John Beutler

**Affiliations:** 1 School of Pharmaceutical Sciences, Sun Yat-sen University, Guangzhou 510006, China; E-Mails: liux66@mail2.sysu.edu.cn (X.L.) zhangjsh0814@163.com (J.Z.); 15521123303@163.com (Q.L.); tanggh5@mail.sysu.edu.cn (G.T.); whongsh@mail.sysu.edu.cn (H.W.); 2 East China Sea Fisheries Research Institute, Chinese Academy of Fishery Sciences, Shanghai 200090, China; E-Mail: chengqifan92@pku.org.cn

**Keywords:** *Sarcophyton elegans*, cembranoids, antimigratory activity

## Abstract

Four new cembranoids, sarcophelegans A–D (**1**–**4**) and six known analogues (**5**–**10**) were isolated from the South China Sea soft coral *Sarcophyton elegans*. Their structures were elucidated through detailed spectroscopic analysis, and the absolute configuration of **1** was confirmed by single-crystal X-ray diffraction. The antimigratory potential of compounds **1**–**10** were evaluated and compounds **2** and **6** were found to inhibit human breast tumor MDA-MB-231 cell migration at 10 μM.

## 1. Introduction

Cembranoids are a group of highly functionalized dierpenoids with a 14-membered carbon ring, an isopropyl residue, and three methyls [1]. Since the first representative, cembrene, was isolated from the pine tree *Pinus albicaulis* Engelm in 1962, hundreds of cembranoids have been reported from plants, insects, alligators, and especially from marine organisms [1,2]. Although cembranoids are indisputably not uniquely marine, their striking presence in soft corals, especially in the genus *Sarcophyton* (Alcyoniidae) [3,4], outstrips their occasional occurrence in other taxa. In recent years, the significant biological activity of cembranoids in terms of antimicrobial, anti-cancer and anti-inflammation effects, together with their fascinating architectures, have attracted great interest from natural product [5,6] and pharmaceutical chemists [7,8].

**Figure 1:**
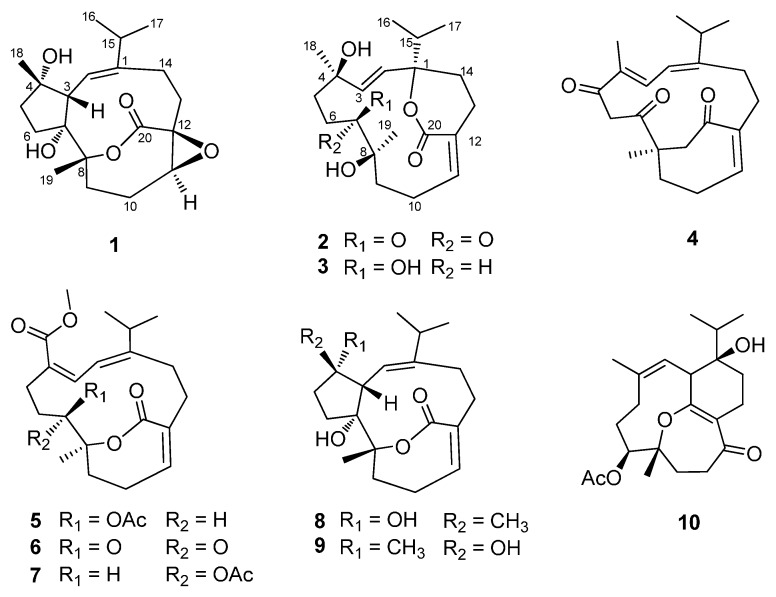
Compounds **1**–**10** isolated from *Sarcophyton elegans*.

The soft coral species of the genus *Sarcophyton* are widely distributed along tropic and subtropic oceans. So far, around 30 species of this genus from different locations have been chemically examined [9]. Previous chemical investigations of *S. elegans* have led to the isolation of several cembranoids, tetracyclic diterpenoids, steroids, and carotenoids [9,10,11,12,13,14,15,16]. As part of our continuing efforts to discover structurally intriguing and bioactivity-significant metabolites from South China Sea marine invertebrates [17,18,19], we undertook a detailed chemical analysis of *S. elegans*, collected in the Xisha Islands, South China Sea, which led to the isolation of four new cembranoids (**1**–**4**) and six known compounds (**5**–**10**) (Figure 1). The antimigratory potential of compounds **1**–**10** was evaluated and compounds **2** and **6** were found to inhibit human breast tumor MDA-MB-231 cell migration at 10 μM. Herein, details of the isolation, structure elucidation, and antimigratory activity of these compounds are described.

## 2. Results and Discussion

### 2.1. Structural Elucidation of New Compounds

The soft coral of *S. elegans* (1 kg, wet weight) was freeze-dried, ground, and extracted with a mixture of CH_2_Cl_2_/MeOH (*v*/*v*, 1:1) at room temperature. After removal of solvent *in vacuo*, the residue was suspended in H_2_O and then partitioned sequentially with petroleum ether (PE) and EtOAc. Various column chromatographic separations of the EtOAc extract afforded compounds **1**–**10**.

**Figure 2:**
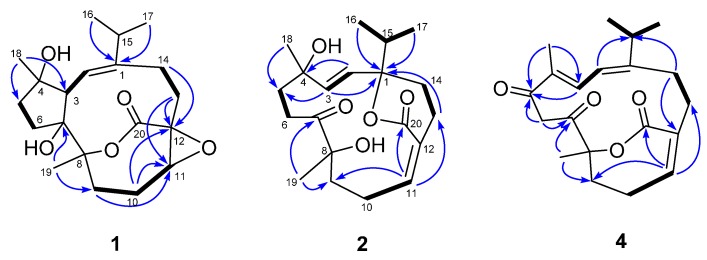
Key ^1^H-^1^H COSY (▬) and HMBC (→) correlations for **1**, **2**, and **4**.

**Figure 3:**
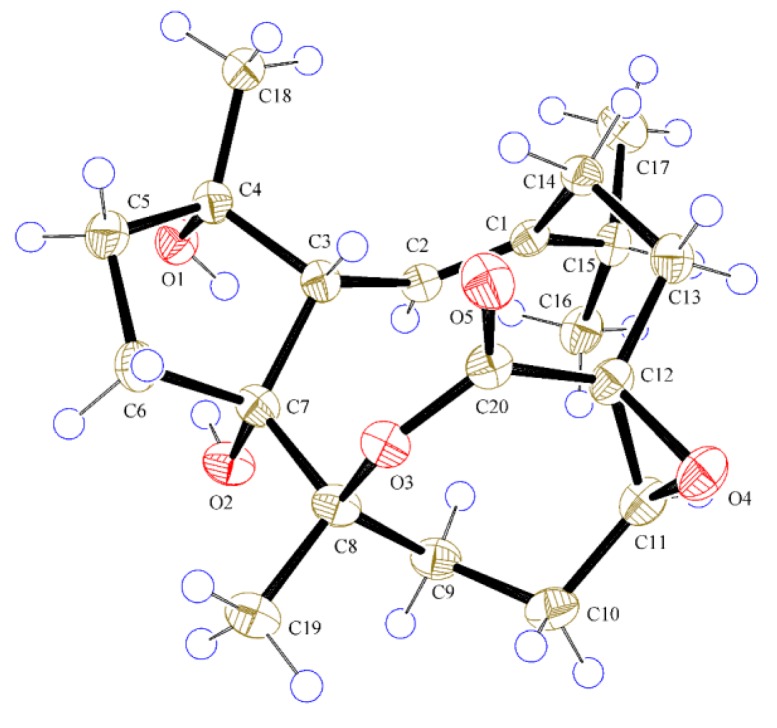
ORTEP depiction for X-ray crystal structures of **1**.

Compound **1**, a colorless crystal, had the molecular formula C_20_H_30_O_5_, as determined by HRESIMS at *m*/*z* 333.2059 [M − H_2_O + H]^+^ (calcd 333.2066), corresponding to six degrees of unsaturation. The IR spectrum exhibited absorption bands for hydroxyl (3451 cm^−1^) and carbonyl (1716 cm^−1^) functionalities. The ^1^H-NMR data (Table 1) of **1** showed two methyl singlets [δ_H_ 1.10 (3H, s, CH_3_-18) and 1.34 (3H, s, CH_3_-19)], an isopropyl group [δ_H_ 1.09 (3H, d, *J* = 6.9 Hz, CH_3_-17), 1.19 (3H, d, *J* = 6.9 Hz, CH_3_-16), and 2.34 (1H, m, H-15)], an oxygenated methine [δ_H_ 3.13 (1H, dd, *J* = 7.0, 7.0 Hz, H-11)], an olefinic proton [δ_H_ 5.69 (1H, d, *J* = 11.3 Hz, H-2)], and a series of aliphatic methylene multiplets. The ^13^C-NMR data (Table 2), in combination with DEPT experiments, resolved 20 carbon resonances attributable to an ester carbonyl group (δ_C_ 173.9), a trisubstituted double bond (δ_C_ 120.1, 147.1), four sp^3^ oxygenated quaternary carbons, three sp^3^ methines (one oxygenated), six sp^3^ methylenes, and four methyls. The above-mentioned data implied that **1** possessed most of the structural features of cembranoid diterpenes, which showed high similarity to those of co-isolated sarsolilide B (**8**) [20]. In comparison with **8**, the signals for Δ^11^ in **8** were replaced by the signals for an epoxy in **1** [δ_H_ 3.13 (1H, dd *J* = 7.0, 7.0 Hz); δ_C_ 65.6 (CH) and 60.4 (C)], indicating that **1** was an 11,12-epoxy derivative of **8**. This was confirmed by HMBC correlations from both H-10 and H-13 to C-11 and C-12, as well as the downfield-shifted carbonyl at C-20 (δ_C_ 173.9 in **1**; δ_C_ 170.3 in **8**) (Figure 2). The relative configuration of **1** was assigned to be the same as that of **8** by comparing their 1D NMR and NOESY data. In particular, the NOESY correlation between H-11 and H-13a indicated the epoxy ring was *cis*-oriented (Figure S6). Finally, the successful performance of the X-ray crystallographic analysis using anomalous scattering of Cu Kα radiation verified the proposed structure and also allowed unambiguous assignment of the absolute configuration of **1** as drawn in Figure 3. Thus, compound **1** was determined as depicted and given the trivial name sarcophelegan A.

**Table 1:** ^1^H-NMR spectroscopic data of **1**–**4** (400 MHz, *J* in Hz, δ in ppm).

Position	1 ^a^	2 ^a^	3 ^a^	4 ^b^
2	5.69, d (11.3)	5.55, d (16.7)	5.54, d (16.3)	6.23 d (11.4)
3	2.35, m (overlapped)	5.69, d (16.7)	5.74, d (16.3)	6.72, brd (11.4)
5a	1.98, m	1.77, m (overlapped)	1.83, m (overlapped)	
5b	1.87, m	1.55, ddd (14.2, 10.5, 7.4)	1.46, m	
6a	2.21, m	3.04, ddd (20.9, 10.5, 7.4)	1.70, m	4.90, d (16.8)
6b	1.90, m	2.44, m (overlapped)	1.38, m	3.29, d (16.8)
7			3.25, brd (11.0, 1.4)	
9a	2.82, dd (14.4, 14.4)	1.96, m	1.88, m	2.40, m (overlapped)
9b	1.73, dd (14.4, 7.6)	1.77, m (overlapped)	1.79, m	2.01, m
10a	2.31, m	3.40, m	3.44, m	2.73, m
10b	1.38, m	2.17, m	2.05, m	2.31, m
11	3.13, dd (7.0, 7.0)	5.67, m	6.19, dd (10.1, 5.1)	6.10, dd (4.1, 4.1)
13a	2.62, m	2.45, m (overlapped)	2.60, m	3.19, m
13b	1.30, m		2.55, m	1.92, m
14a	2.51, m	2.12, m	2.16, m	2.56, dd (13.8, 13.8)
14b	2.12, m	1.76 m	1.84, m (overlapped)	2.23, dd (13.8, 7.8)
15	2.34, m (overlapped)	1.86, m	1.87, m	2.41, m (overlapped)
16	1.19, d (6.9)	0.98, d (6.8)	0.98, d (7.1)	1.07, d (6.8)
17	1.09, d (6.9)	0.95, d (6.8)	0.96, d (7.1)	1.09, d (6.8)
18	1.10, s	1.39, s	1.33, s	1.83, s
19	1.34, s	1.21, s	1.15, s	1.54, s

^a^ Measured in CD_3_OD; ^b^ Measured in CDCl_3_.

**Table 2:** ^13^C-NMR spectroscopic data of **1**–**4** (100 MHz, δ in ppm).

Position	1 ^a^	2 ^a^	3 ^a^	4 ^b^
1	147.1, C	87.8, C	88.0, C	158.8, C
2	120.1, CH	128.2, CH	128.3, CH	119.4, CH
3	52.0, CH	140.4, CH	140.8, CH	137.8, CH
4	83.4, C	72.8, C	73.4, C	133.3, C
5	38.5, CH_2_	36.5, CH_2_	41.0, CH_2_	195.1, C
6	34.7, CH_2_	35.2, CH_2_	25.4, CH_2_	45.7, CH_2_
7	88.9, C	219.3, C	76.0, CH	204.3, C
8	91.9, C	79.7, C	75.8, C	86.1, C
9	29.9, CH_2_	42.0, CH_2_	39.0, CH_2_	33.3, CH_2_
10	24,1, CH_2_	25.9, CH_2_	25.5, CH_2_	27.2, CH_2_
11	65.6, CH	150.3, CH	149.5, CH	143.6, CH
12	60.4, C	125.1, C	125.5, C	130.9, C
13	33.0, CH_2_	25.0, CH_2_	25.8, CH_2_	37.1, CH_2_
14	25.8, CH_2_	27.5, CH_2_	27.5, CH_2_	27.6, CH_2_
15	34.0, CH	38.4, CH	38.9, CH	36.3, CH
16	21.3, CH_3_	17.4, CH_3_	17.4, CH_3_	22.4, CH_3_
17	23.9, CH_3_	17.1, CH_3_	17.1, CH_3_	21.7, CH_3_
18	26.2, CH_3_	29.3, CH_3_	30.2, CH_3_	10.9, CH_3_
19	25.1, CH_3_	28.5, CH_3_	24.4, CH_3_	28.9, CH_3_
20	173.9, C	168.8, C	169.5, C	165.8, C

^a^ Measured in CD_3_OD; ^b^ Measured in CDCl_3_.

Compound **2** possessed a molecular formula of C_20_H_30_O_5_ as determined by HRESIMS at *m*/*z* 373.1986 [M + Na]^+^, which was compatible with its 1D NMR data. The ^1^H-NMR data of **2** (Table 1) showed signals for two methyl singlets [δ_H_ 1.21, (3H, s, CH_3_-19) and 1.39 (3H, s, CH_3_-18)], an isopropyl group [δ_H_ 0.95 (3H, d, *J* = 6.8 Hz, CH_3_-17), 0.98 (3H, d, *J* = 6.8 Hz, CH_3_-16), and 1.86 (1H, m, H-15)], two *trans*-olefinic protons [δ_H_ 5.55 (1H, d, *J* = 16.7 Hz, H-2) and 5.69 (1H, d, *J* = 16.7 Hz, H-3)], an olefinic proton [δ_H_ 5.67 (1H, m, H-11)], and a series of aliphatic methylene multiplets. The 20 carbon resonances were classified by DEPT experiments as a ketone carbonyl group (δ_C_ 219.3), an ester carbonyl group (δ_C_ 168.8), two double bonds (δ_C_ 125.1, 128.2, 140.4, and 150.3), three sp^3^ oxygenated quaternary carbons, a sp^3^ methine, six sp^3^ methylenes, and four methyls (Figure S8). The above-mentioned information was similar to that of sartrolide E [21]. However, analysis of HSQC and HMBC data (Figures S10 and S11) revealed that the chemical shift of C-1 (δ_C_ 76.7) in sartrolide E was downfield-shifted to δ_C_ 87.8 in **2**, while C-8 was upfield-shifted from δ_C_ 87.0 to δ_C_ 79.7 (Figure 2), indicating that the linkage of the lactone ring from C-12 to C-8 in sartrolide E was migrated to C-1 in **2**. This was further supported by comparison of the C-1 and C-8 chemical shifts of **2** with those of a known analogue, laevigatlactone E [22], sharing the similar lactone linkage as that in **2** (δ_C_ 87.9, C-1 and δ_C_ 73.2, C-8, in laevigatlactone E).

**Figure 4:**
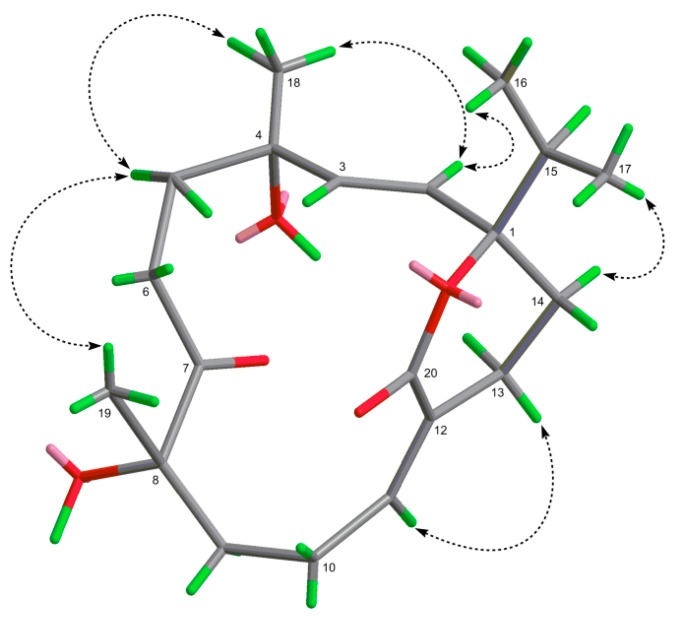
Selected NOESY correlations of **2** (↔).

The relative configuration of **2** was determined on the basis of the NOESY experiment (Figure S12). The NOESY correlation observed between H-11 and H-13 suggested the *E* geometry for the Δ^11^. The crucial NOE correlations between H-2/CH_3_-16 and H-2/CH_3_-18 revealed that the isopropyl group and CH_3_-18 were co-facial and were arbitrarily designated as α-oriented, while the interactions of H-5a with CH_3_-18 and CH_3_-19 indicated that the CH_3_-18 and CH_3_-19 were both α-oriented (Figure 4). Thus, compound **2** was deduced as shown and named sarcophelegan B.

Compound **3** displayed the HRESIMS ion at *m*/*z* 375.2147 [M + Na]^+^, consistent with a molecular formula of C_20_H_32_O_5_, two mass units more than that of **2**. The ^1^H and ^13^C NMR data of **3** (Table 1 and Table 2) were very similar to those of **2** except for the presence of an additional oxygenated methine (δ_H_ 3.25; δ_C_ 76.0) in **3** instead of the ketone group (δ_C_ 219.3, C-7) in **2**, indicating that **3** was a 7-hydrogenated derivative of **2**. HMBC correlation from CH_3_-19 and H_2_-5 to the oxygenated methine (δ_C_ 76.0) confirmed the location of the hydroxyl group at C-7. This was also supported by the upfield-shifted signals of C-6 and C-8 in **3** with respect to those in **2** (δ_C_ 25.4, C-6; 75.8, C-8 in **3**; δ_C_ 35.2, C-6; 79.7, C-8 in **2**). The relative configurations at C-1, C-4, and C-8 of **3** were assigned to be the same as those of **2** by comparing their 1D NMR and NOESY data. The 7-OH was designated as β by the NOE correlation between H-7 and CH_3_-19, as in the Chem3D molecular modeling study, the 7β-OH isomer of **3** display a distance of 2.573 Å between H-7 and CH_3_-19, while the 7α-OH isomer showed a large distance of 3.738 Å (Figure S25). Thus, compound **3** was deduced as shown and named sarcophelegan C.

Compound **4**, a colorless oil, exhibited a molecular formula of C_20_H_26_O_4_ as determined by HRESIMS and the data of ^13^C-NMR. The ^1^H- and ^13^C-NMR spectra of **4** (Figures S19 and S20) showed signals for two ketone signals (δ_C_ 195.1 and 204.3), an α,β-unsaturated-ε-lactone (δ_C_ 86.1, 130.9, 143.6, and 165.8), an isopropyl group [δ_C_ 21.7, 22.4, and 36.3; δ_H_ 1.07 (3H, d, *J* = 6.8 Hz, 1.09 (3H, d, *J* = 6.8 Hz)), and 2.41 (1H, m)], two double bonds [δ_C_ 119.4, 133.3, 137.8, and 158.8; δ_H_ 6.23 (1H, d, *J* = 11.4 Hz) and 6.72 (1H, brd, *J* = 11.4 Hz)], and two methyl singlets [δ_C_ 10.9 and 28.9; δ_H_ 1.54 (3H, s) and1.83 (3H, s)]. These data showed high similarity to those of (1*Z*,5*S*,9*E*,11*E*)-5,9-dimethyl-12-isopropyl-6-oxocyclotetradeca-1,9,11-triene-1,5-carbolactone [23], a cembranoid diterpene previously reported from the same genus, except for the presence of an additional carbonyl group (δ_C_ 195.1), which indicated that **4** was a carbonylated derivative of (1*Z*,5*S*,9*E*,11*E*)-5,9-dimethyl-12-isopropyl-6-oxocyclotetradeca-1,9,11-triene-1,5-carbolactone. HMBC correlations from CH_3_-18 and H-3 to the carbonyl carbon revealed that the carbonyl group was located at C-5. This was further supported by the downfield-shifted H-3 signal in **4** with respect to that in (1*Z*,5*S*,9*E*,11*E*)-5,9-dimethyl-12-isopropyl-6-oxocyclotetradeca-1,9,11-triene-1,5-carbolactone (δ_H_ 6.72 in **2**; δ_H_ 6.07 in (1*Z*,5*S*,9*E*,11*E*)-5,9-dimethyl-12-isopropyl-6-oxocyclotetradeca-1,9,11-triene-1,5-carbolactone). Detailed 2D NMR analyses [^1^H-^1^H COSY, HSQC, and HMBC (Figures S21-23)] permitted the establishment of the gross structure of **4** as depicted in Figure 2. The absolute configuration of the only chiral center C-8 in **4** was proposed as *S* based on comparison of its specific rotation (${\left[\alpha \right]}_{D}^{20}$ + 190.8) with (1*Z*,5*S*,9*E*,11*E*)-5,9-dimethyl-12-isopropyl-6-oxocyclotetradeca-1,9,11-triene-1,5-carbolactone (${\left[\alpha \right]}_{D}^{20}$ + 177), which was also supported by the biogenetic origin of this skeleton. Interestingly, cembranoids with C-3 and C-7 cyclization exclusively give *R* configuration at C-8 [20]. Compound **4** was given the trivial name sarcophelegan D.

The known compounds emblide (**5**) [4], ketoemblide (**6**) [24], sarcrassin D (**7**) [4], sarsolilides B (**8**) [20], sarsolilide C (**9**) [20], and dihydrosarsolenone (**10**) [20] were identified by comparison of their NMR and MS data with those in the literature.

### 2.2. Antimigratory Activity

Metastasis is one of the major biological characteristics of cancer cells. The wound-healing assay is a simple and widely used tool to investigate *in vitro* directional cell migration [5,25,26]. The effects of compounds **1**–**10** on the migration of human breast cancer MDA-MB-231 cells were evaluated using wound-healing assays. The ability of the compounds to inhibit the migration of the cancer cells into the wound is measured by comparing the original wound width before assay with the wound width after 48 h incubation [relative wound closure = (W_0_ − W_48_)/W_0_]. The higher antimigratory activity of the compound is, the smaller the wound-relative closure value it generates.

**Figure 5:**
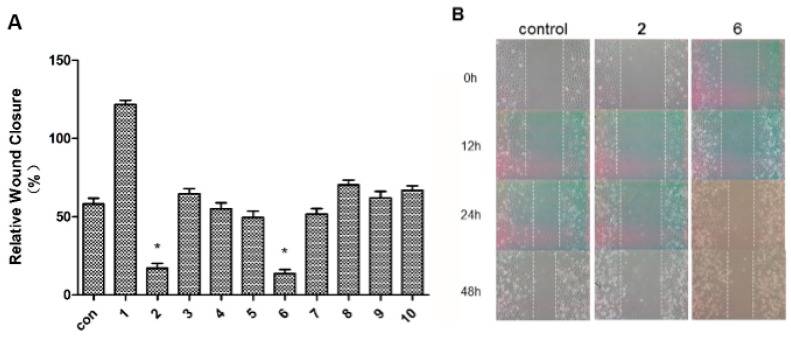
Wound-healing assays on compounds **1**–**10** with human breast tumor cell MDA-MB-231. (**A**) The antimigration effects of **1**–**10** at 10 μM on the tumor cells (* *p* < 0.05); (**B**) The incubation of **2** and **6** with tumor cells at 0, 12, 24, and 48 h (left: control; middle: **2** at 10 μM; right: **6** at 10 μM).

Among **1**–**10**, compounds **2** and **6** had the greatest capability to inhibit the migration of MDA-MB-231 cells while others did not show evident activity in comparison with control. Furthermore, compounds **2** and **6** inhibited the cell migration in a time dependent manner (Figure 5B).

## 3. Experimental Section

### 3.1. General Experimental Procedures

X-ray data were collected using an Agilent Xcalibur Nova X-ray diffractometer (Agilent, Santa Clara, CA, USA). Melting points were measured on an X-4 melting instrument and are uncorrected. Optical rotations were measured on a Perkin-Elmer 341 polarimeter (Perkin-Elmer, Waltham, MA, USA). UV spectra were recorded on a Shimadzu UV-2450 spectrophotometer (Shimadzu, Kyoto, Japan). IR spectra were determined on a Bruker Tensor 37 infrared spectrophotometer (Bruker, Karlsruhe, Germany). NMR spectra were measured on a Bruker AM-400 spectrometer (Bruker, Karlsruhe, Germany) at 25 °C. ESIMS was measured on a Finnigan LCQ Deca instrument (Thermo Finnigan, San Jose, CA, USA), and HRESIMS was performed on a Waters Micromass Q-TOF (Waters, Milford, MA, USA). A Shimadzu LC-20 AT equipped with a SPD-M20A PDA detector (Shimadzu, Kyoto, Japan) was used for HPLC. A YMC-pack ODS-A column (250 ´ 10 mm, S-5 μm, 12 nm) (YMC, Tokyo, Japan) was used for semipreparative HPLC separation. Wound closure was monitored and photographed with a Nikon Eclipse inverted microscope. Silica gel (300−400 mesh, Qingdao Marien Chemical Co., Ltd., Qingdao, Shandong, China), reversed-phase C_18_ (Rp-C_18_) silica gel (12 nm, S-50 μm, YMC Co., Ltd., Kyoto, Japan), Sephadex LH-20 gel (Amersham Biosciences, Piscataway, NJ, USA), and MCI gel (CHP20P, 75−150 μm, Mitsubishi Chemical Industries Ltd. Tokyo, Japan) were used for column chromatography (CC). All solvents used were of analytical grade (Guangzhou Chemical Reagents Co., Ltd., Guangzhou, China).

### 3.2. Animal Material

The soft coral *S. elegans* were collected from the Xisha Islands in the South China Sea, in October 2014, at a depth of 8–10 m of water. The biological material was frozen immediately until used and was identified by Cheng-Qi Fan from East China Sea Fisheries Research Institute. A voucher specimen (accession number: HLRZ201410) has been deposited at the School of Pharmaceutical Sciences, Sun Yat-sen University, Guangzhou, China.

### 3.3. Extraction and Isolation

The frozen samples (1 kg, wet weight) were extracted with CH_2_Cl_2_/MeOH (1:1, 3 × 1 L) at room temperature. After removal of solvent in vacuo, the residue (16 g) was suspended in H_2_O (200 mL) and partitioned sequentially to give dried petroleum ether (2 g) and EtOAc (4 g) extracts. The EtOAc extract was subjected to silica gel column chromatography eluted with a CH_2_Cl_2_/MeOH gradient (100:1→10:1) to afford five fractions (I–V). Fr. II (460 mg) was subjected to Rp-C_18_ silica gel CC eluted with MeOHH_2_O (6:4 to 10:0), followed by a Sephadex LH-20 and eluted with EtOH to afford **2** (11 mg), **5** (72 mg), **6** (45 mg), and **7** (3.7 mg). Fr. III (1.4 g) was chromatographed over Sephadex LH-20 (CH_2_Cl_2_/MeOH, v/v, 1:1), followed by Rp-C_18_ silica gel eluted with a CH_3_CN/H_2_O gradient (5:5→10:0) to obtain four sub-fractions (Fr. IIIa–IIId). Fr. IIIb was further separated by HPLC equipped with an ODS-18 column using CH_3_CN/H_2_O (65:35, *v*/*v*; 3 mL·min^−1^) to afford **1** (8.1 mg, *t*_R_ 8.5 min), **8** (6 mg, *t*_R_ 11 min), and **9** (18 mg, *t*_R_ 13.5 min). Fr. IIId was purified by repeating the HPLC conditions described above to yield **3** (41 mg, *t*_R_ 13 min) and **4** (3.7 mg, *t*_R_ 17 min). Fr. IIIc was chromatographed with silica gel CC (CH_2_Cl_2_/MeOH, 40:1) to give **10** (25.2 mg).

### 3.4. Cell Culture

Human breast tumor cells (MDA-MB-231) were obtained from the Institute of Chinese Academy of Medical Sciences, Beijing, China. MDA-MB-231 cells were cultured in RPMI-1640 containing 10% FBS in cell culture flasks under a humidified 5% CO_2_ and 95% air atmosphere at 37 °C.

### 3.5. Wound-Healing Assays

The method used to detect migration by wound-healing assay was previously described [5,25,26]. Briefly, the cells were allowed to grow to 90% confluence in 6-well plates. Once the monolayer was developed, a wound was made by scrapping with a 100 μL pipet tip to create a denuded zone (gap) of constant width. Subsequently, cellular debris was washed with 2‰ FBS, and the MDA-MB-231 cells were exposed to 10 μM of compounds **1**–**10**. Wound closure was monitored and photographed at 0, 12 h, 24 h, and 48 h with a Nikon Eclipse inverted microscope. Wound width was measured immediately before (W_0_) and after the 48 h (W_48_) incubation. To quantify the migrated cells, pictures of the initial wounded monolayers were compared with the corresponding pictures of cells at the end of the incubation. Artificial lines fitting the cutting edges were drawn on pictures of the original wounds and overlaid on the pictures of cultures after incubation. Figure 5A represents wound closure values for different compounds (**1**–**10**), relative to the control (time 0).

### 3.6. Statistical Analysis

Data were expressed as the mean ± SD of at least three independent experiments. To compare three or more groups, one-way analysis of variance (ANOVA) was used followed by Newman-Keuls *post hoc* test. Statistical analysis was performed using GraphPad Prism software (5.01, GraphPad Software Inc., San Diego, CA, USA).

*Sarcopelegan A* (**1**): Colorless crystals; mp 187–189 °C; ${\left[\alpha \right]}_{D}^{20}$ +16.7 (*c* 0.23, MeOH); UV (MeOH) λ_max_ (log ε) 208.4 (6.82) nm; IR (KBr) ν_max_ 3451, 2958, 2925, 1716, 1237 cm^−1^; ^1^H- and ^13^C-NMR data see Table 1 and Table 2; HRESIMS *m*/*z* 333.2059 (calcd for C_20_H_29_O_4_ [M − H_2_O + H]^+^, 333.2066).

*Sarcopelegan B* (**2**): Colorless oil; ${\left[\alpha \right]}_{D}^{20}$ −3.7 (*c* 0.46, MeOH); UV (MeOH) λ_max_ (log ε) 230.4 (6.82) nm; IR (KBr) ν_max_ 3396, 2933, 1699, 1381, 983 cm^−1^; ^1^H- and ^13^C-NMR data see Table 1 and Table 2; HRESIMS *m*/*z* 373.1986 (calcd for C_20_H_30_O_5_Na [M + Na]^+^, 373.1991).

*Sarcopelegan C* (**3**): Colorless oil; ${\left[\alpha \right]}_{D}^{20}$ −10.0 (*c* 0.14, MeOH); UV (MeOH) λ_max_ (log ε) 232.2 (7.17), 210.0 (7.05) nm; IR (KBr) ν_max_ 3357, 2966, 2929, 1694, 1071 cm^−1^; ^1^H- and ^13^C-NMR data see Table 1 and Table 2; HRESIMS *m*/*z* 375.2147 (calcd for C_20_H_32_O_5_Na [M + Na]^+^, 375.2143).

*Sarcopelegan D* (**4**): Colorless oil; ${\left[\alpha \right]}_{D}^{20}$ +190.8 (*c* 0.20, MeOH); UV (MeOH) λ_max_ (log ε) 298.2 (7.10), 219.8 (6.98) nm; IR (KBr) ν_max_ 3454, 2961, 2067, 1621, 1270 cm^−1^; ^1^H- and ^13^C-NMR data see Table 1 and Table 2; HRESIMS *m*/*z* 331.1901 (calcd for C_20_H_27_O_4_ [M + H]^+^, 331.1909).

*Crystal data for compound* (**1**): C_20_H_30_O_5_, M = 350.44, 0.5 × 0.1 × 0.2 mm^3^, space group *P*6_5_ (No. 170), *V* = 2769.03(4) Å^3^, *Z* = 6, *D*_c_ = 1.261 g·cm^−3^, *F*_000_ = 1140, Xcalibur, Onyx, Nova, Cu Kα radiation, λ = 1.54184 Å, *T* = 293(2) K, 2θ_max_ = 143.5°, 35758 reflections collected, 3601 unique (R_int_ = 0.0507). Final *GooF* = 1.043, *R_1_* = 0.0285, *wR_2_* = 0.0757, *R* indices based on 3523 reflections with I > 2 sigma (I) (refinement on *F*^2^), 232 parameters, 1 restraint. Lp and absorption corrections applied, μ = 0.723 mm^−1^. Flack parameter = −0.02 (11). Crystallographic data for the structure of **1** have been deposited in the Cambridge Crystallographic Data Centre (deposition number: CCDC 1401385). The data can be obtained free of charge via http://www.ccdc.cam.ac.uk/conts/retrieving.html (or from the CCDC, 12 Union Road, Cambridge CB2 1EZ, UK; Fax: +44 1223 336033; E-mail: deposit@ccdc.cam.ac.uk).

## 4. Conclusions

Four new cembranoids and six known analogues were isolated from the South China Sea soft coral *S. elegans*, collected from the Xisha Islands. Their structures were elucidated through detailed spectroscopic analysis, and the absolute configuration of **1** was confirmed by single-crystal X-ray diffraction. The antimigratory potential of compounds **1**–**10** were evaluated, two of which were found to inhibit human breast tumor MDA-MB-231 cell migration at 10 μM. The current research not only expanded the members of the cembranoid family, but may also provide some diterpene prototypes for further development of anti-cancer leads with antimigratory properties.

## Supplementary Materials

1D and 2D NMR spectra of **1**–**4** were provided. These materials can be accessed at: http://www.mdpi.com/1420-3049/20/07/13324/s1.
