# Supplementary material for: Bioactive Cembranoids from the South China Sea Soft Coral Sarcophyton elegans
Source: Molecules. 2015 Jul 22;20(7):13324–35. doi: 10.3390/molecules200713324 (PMC6331945; doi:10.3390/molecules200713324)
Supplement: Supplementary file 1 [file molecules-20-13324-s001.pdf]

Supplementary Materials

Contents:

Figure S1. ^{1}H NMR (400 MHz, CD_3OD) spectrum of sarcophelegan A (**1**).
Figure S2. ^{13}C NMR (100 MHz, CD_3OD) spectrum of sarcophelegan A (**1**).
Figure S3. ^{1}H-^{1}H COSY spectrum of sarcophelegan A (**1**) in CD_3OD.
Figure S4. HSQC spectrum of sarcophelegan A (**1**) in CD_3OD.
Figure S5. HMBC spectrum of sarcophelegan A (**1**) in CD_3OD.
Figure S6. NOESY spectrum of sarcophelegan A (**1**) in CD_3OD.
Figure S7. ^{1}H NMR (400 MHz, CD_3OD) spectrum of sarcophelegan B (**2**).
Figure S8. ^{13}C NMR (100 MHz, CD_3OD) spectrum of sarcophelegan B (**2**).
Figure S9. ^{1}H-^{1}H COSY spectrum of sarcophelegan B (**2**) in CD_3OD.
Figure S10. HSQC spectrum of sarcophelegan B (**2**) in CD_3OD.
Figure S11. HMBC spectrum of sarcophelegan B (**2**) in CD_3OD.
Figure S12. NOESY spectrum of sarcophelegan B (**2**) in CD_3OD.
Figure S13. ^{1}H NMR (400 MHz, CD_3OD) spectrum of sarcophelegan C (**3**).
Figure S14. ^{13}C NMR (100 MHz, CD_3OD) spectrum of sarcophelegan C (**3**).
Figure S15. ^{1}H-^{1}H COSY spectrum of sarcophelegan C (**3**) in CD_3OD.
Figure S16. HSQC spectrum of sarcophelegan C (**3**) in CD_3OD.
Figure S17. HMBC spectrum of sarcophelegan C (**3**) in CD_3OD.
Figure S18. NOESY spectrum of sarcophelegan C (**3**) in CD_3OD.
Figure S19. ^{1}H NMR (400 MHz, $CDCl_3$) spectrum of sarcophelegan D (**4**).
Figure S20. ^{13}C NMR (100 MHz, $CDCl_3$) spectrum of sarcophelegan D (**4**).
Figure S21. ^{1}H-^{1}H COSY spectrum of sarcophelegan D (**4**) in $CDCl_3$.
Figure S22. HSQC spectrum of sarcophelegan D (**4**) in $CDCl_3$.
Figure S23. HMBC spectrum of sarcophelegan D (**4**) in $CDCl_3$.
Figure S24. NOESY spectrum of sarcophelegan D (**4**) in $CDCl_3$.
Figure S25. The Chem3D molecular modeling study of **3**.

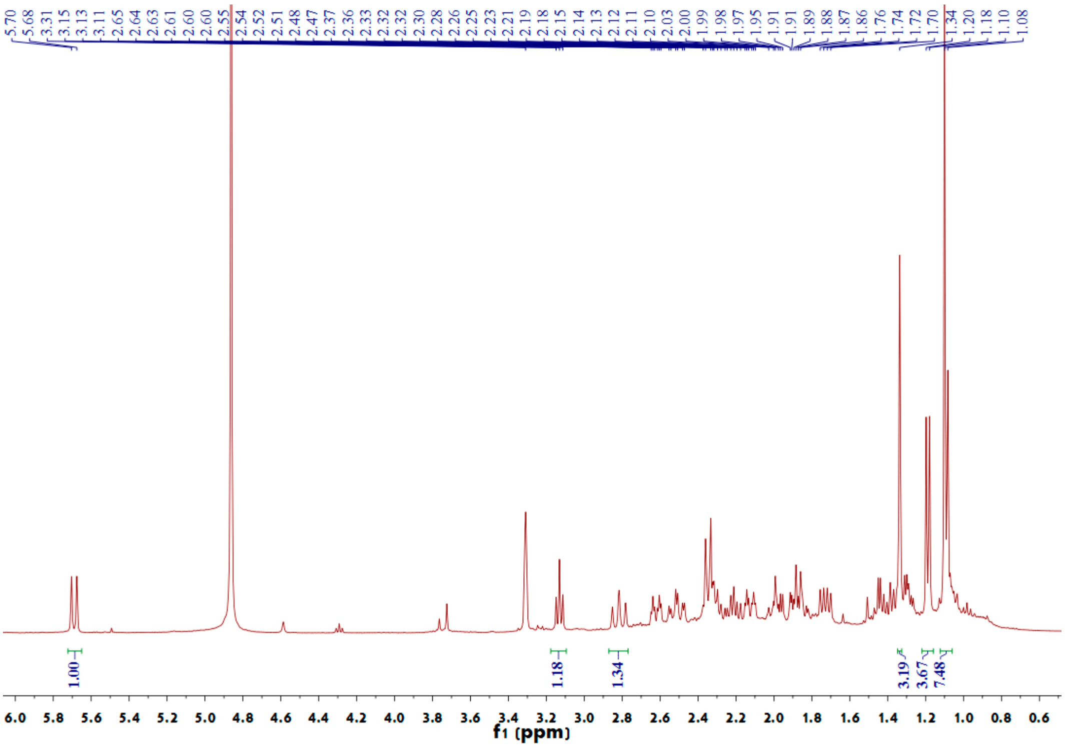

Figure S1. ^{1}H NMR (400 MHz, CD_3OD) spectrum of sarcophelegan A (**1**).

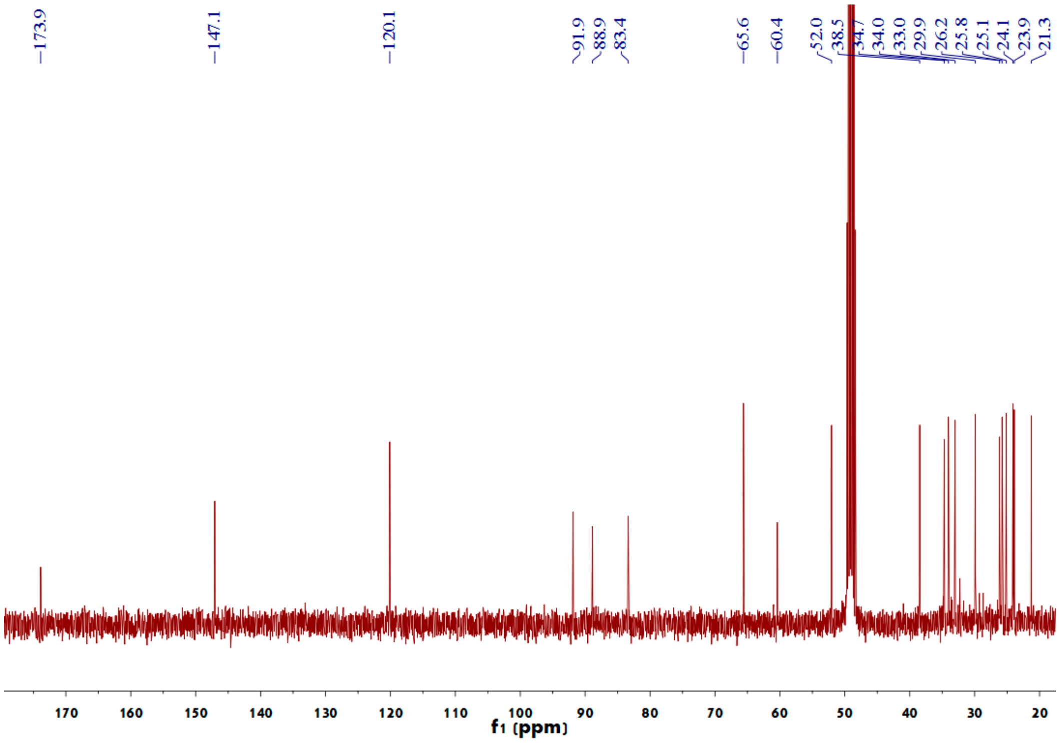

Figure S2. ^{13}C NMR (100 MHz, MeOD) spectrum of sarcophelegan A (**1**).

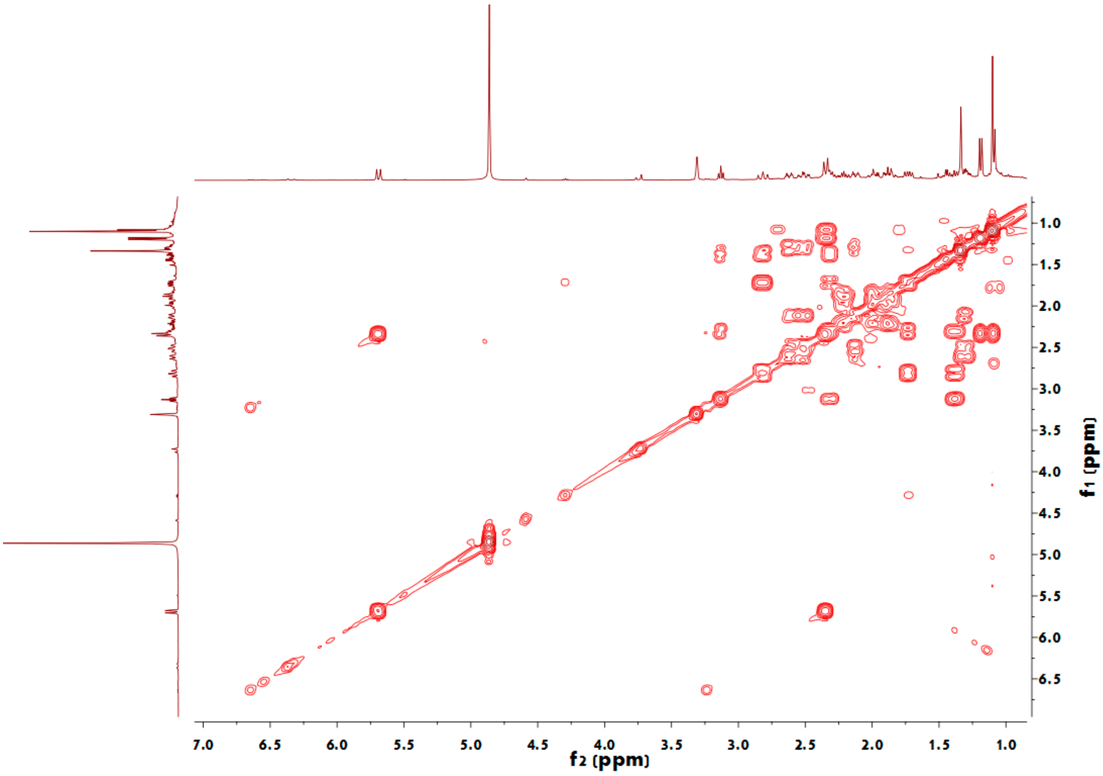

Figure S3. ^{1}H-^{1}H COSY spectrum of sarcophelegan A (**1**) in CD_3OD.

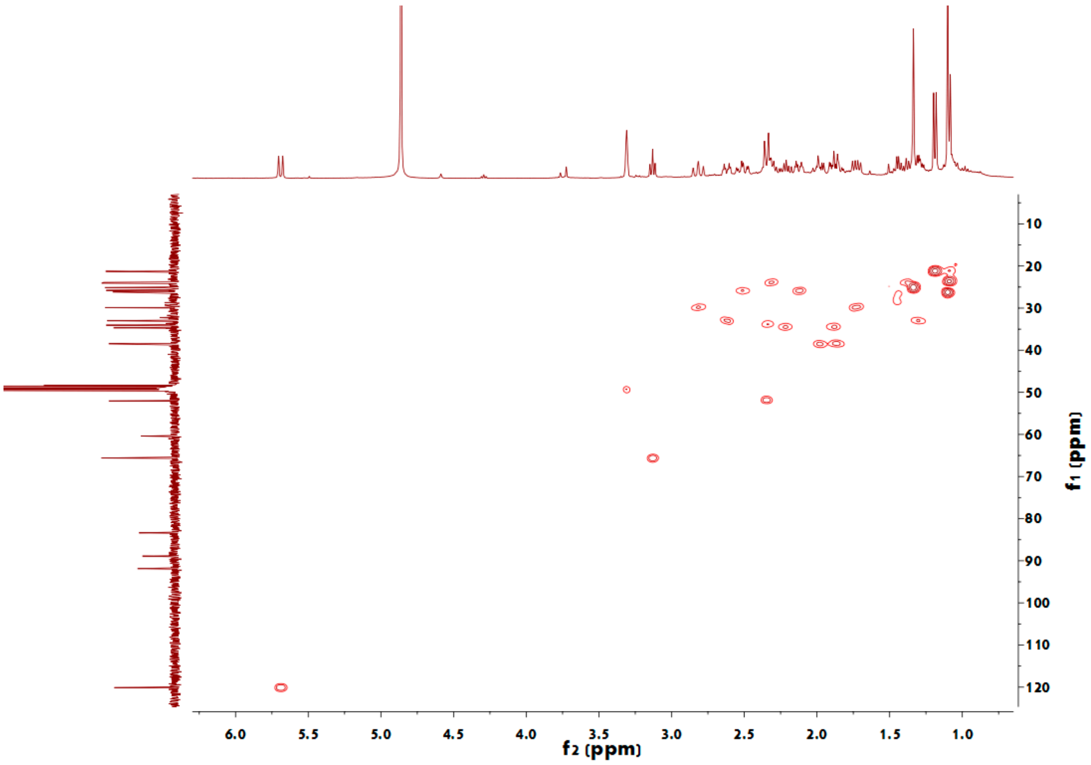

Figure S4. HSQC spectrum of sarcophelegan A (**1**) in CD_3OD.

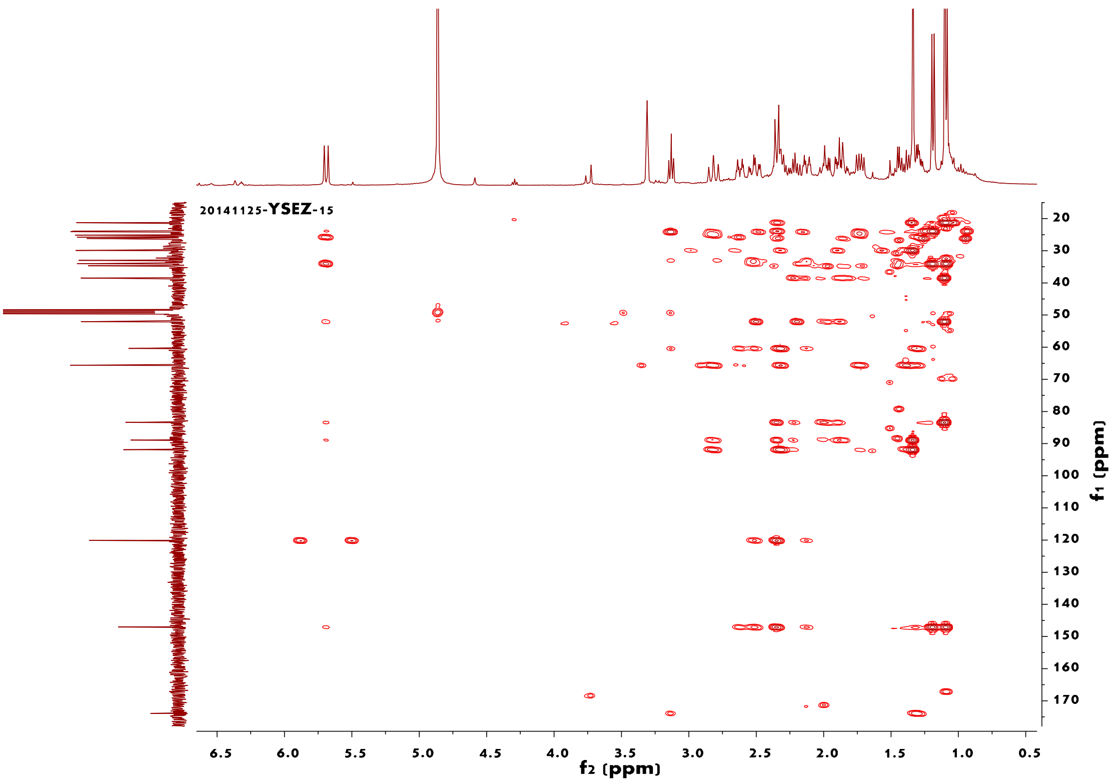

Figure S5. HMBC spectrum of sarcophelegan A (**1**) in CD_3OD.

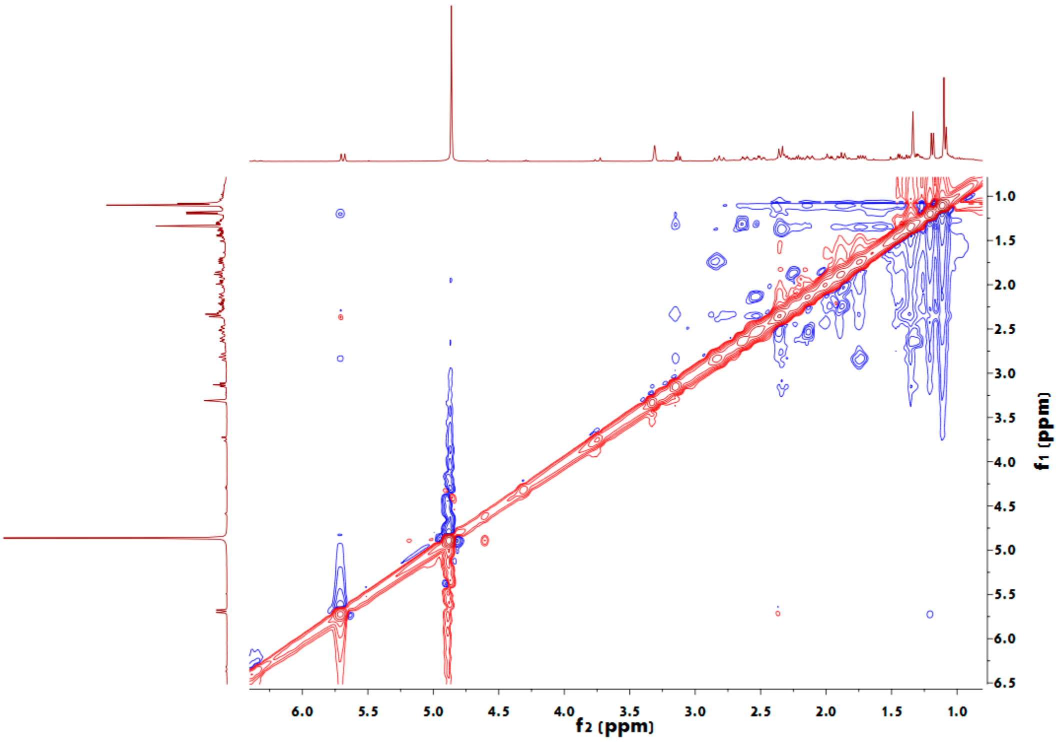

Figure S6. NOESY spectrum of sarcophelegan A (**1**) in CD_3OD.

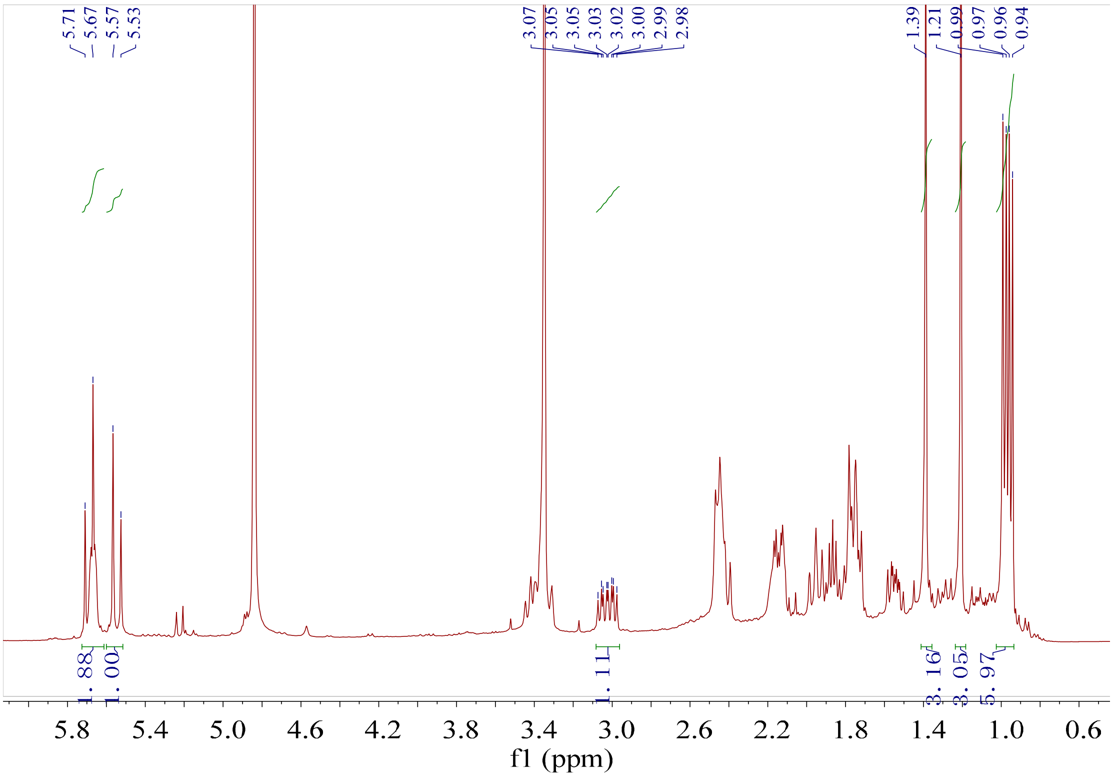

Figure S7. ^{1}H NMR (400 MHz, CD3OD) spectrum of sarcophelegan B (**2**).

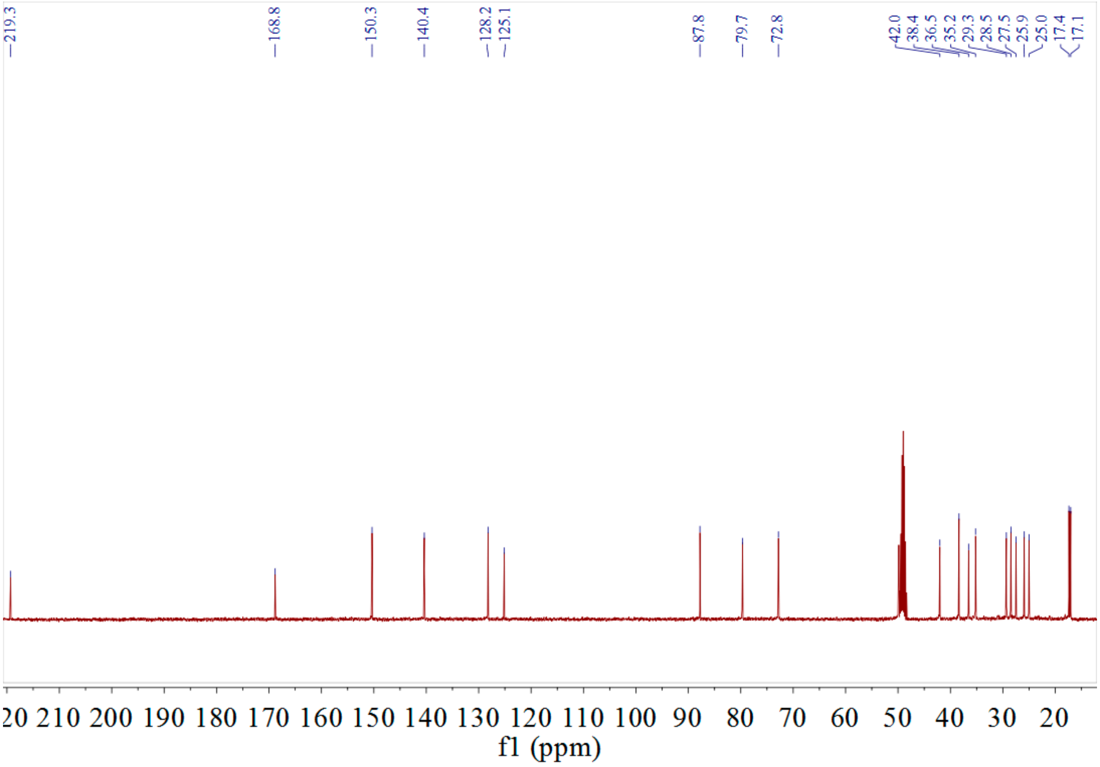

Figure S8. ^{13}C NMR (100 MHz, CD3OD) spectrum of sarcophelegan B (**2**).

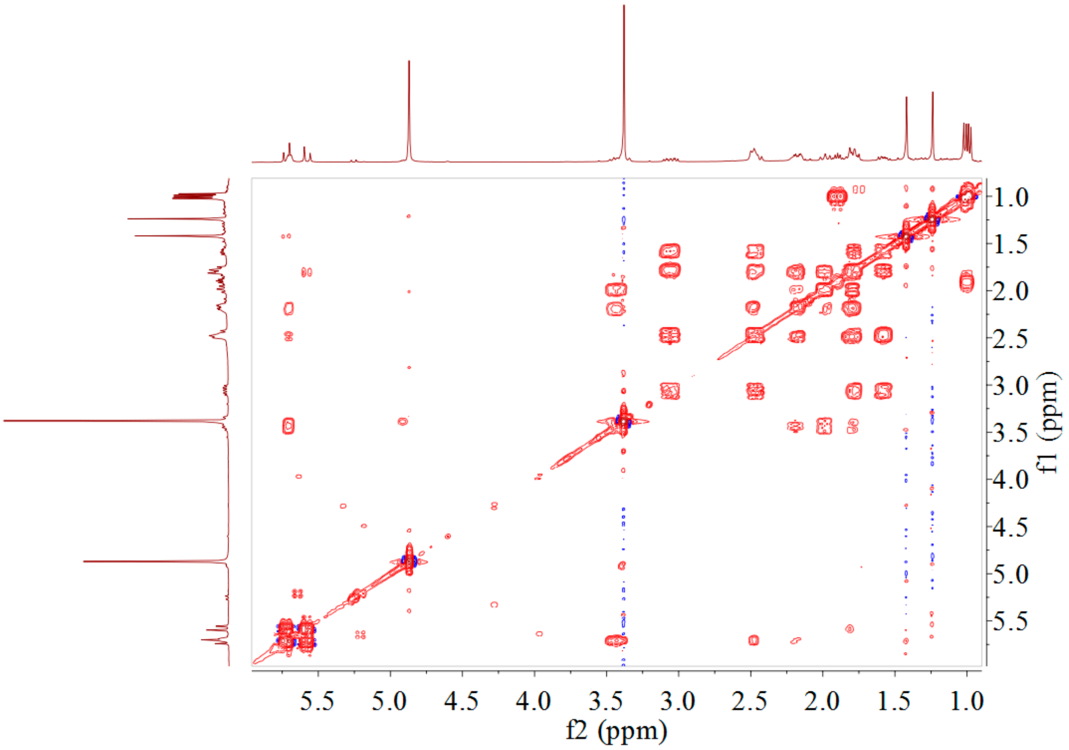

Figure S9. ^{1}H-^{1}H COSY spectrum of sarcophelegan B (**2**) in CD_3OD.

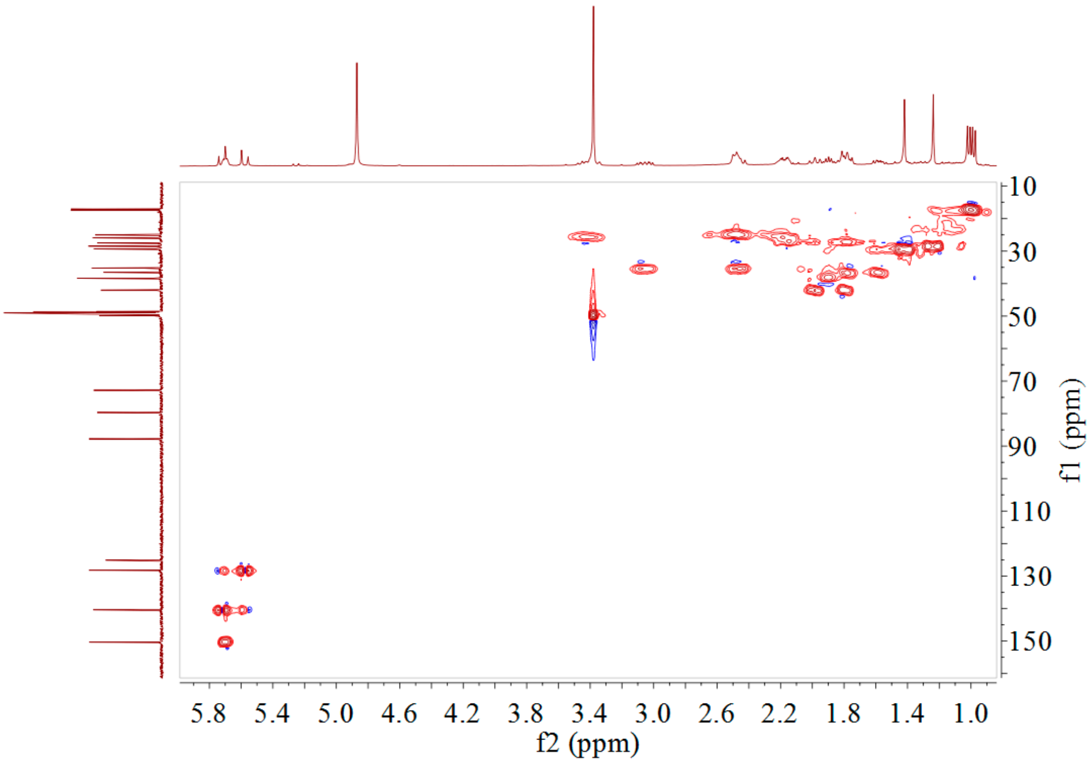

Figure S10. HSQC spectrum of sarcophelegan B (**2**) in CD_3OD.

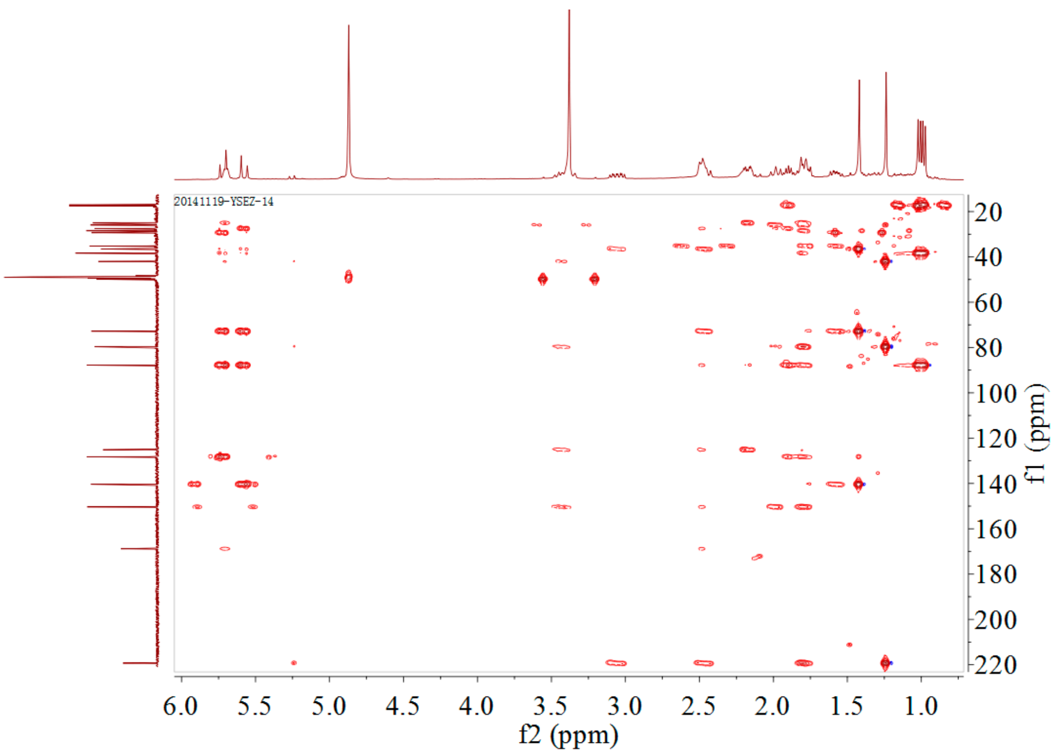

Figure S11. HMBC spectrum of sarcophelegan B (**2**) in CD_3OD.

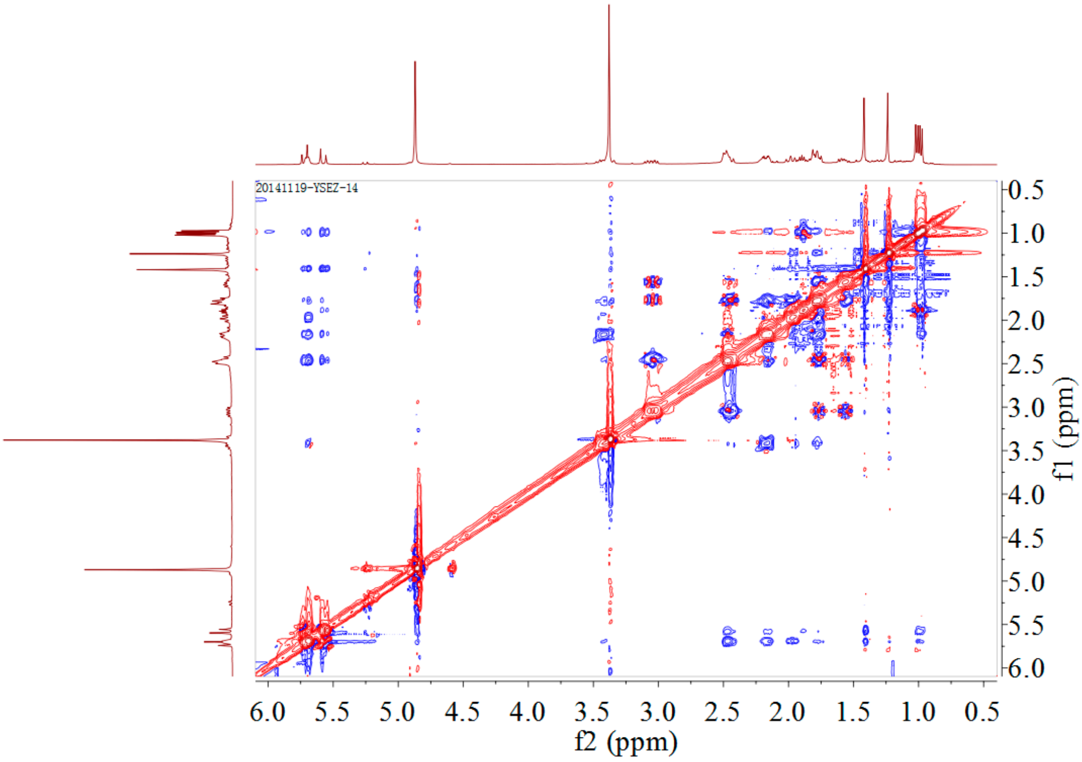

Figure S12. NOESY spectrum of sarcophelegan B (**2**) in CD_3OD.

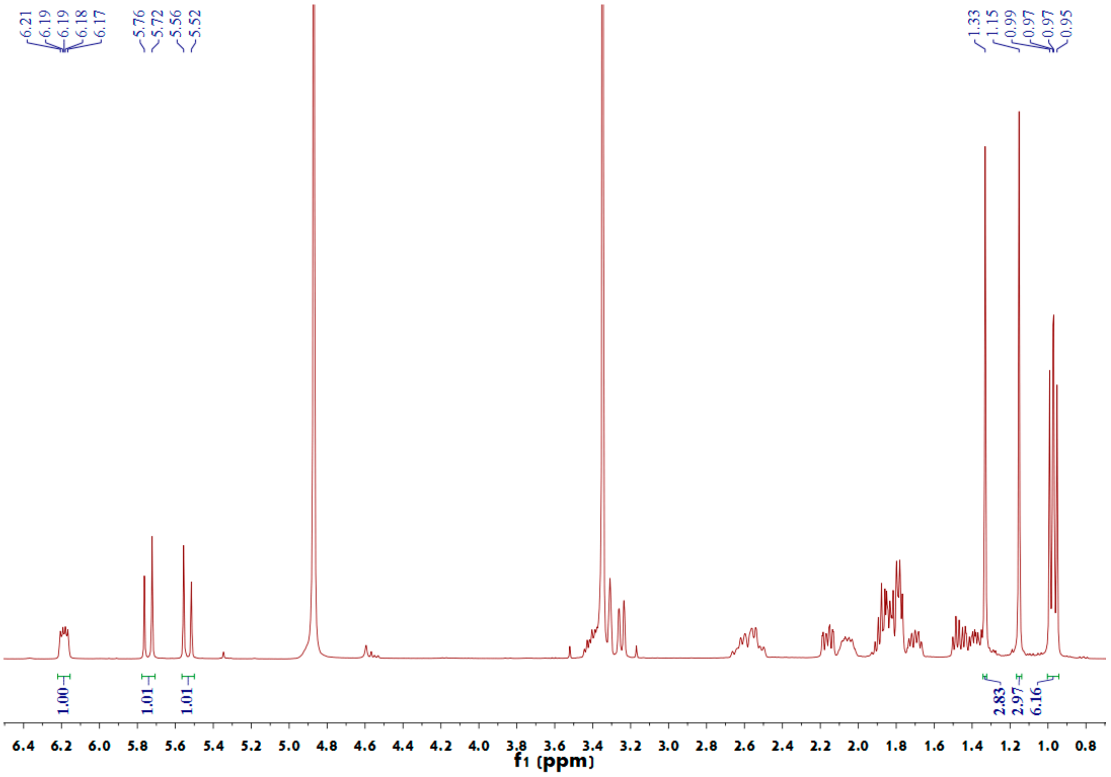

Figure S13. ^{1}H NMR (400 MHz, CD_3OD) spectrum of sarcophelegan C (**3**).

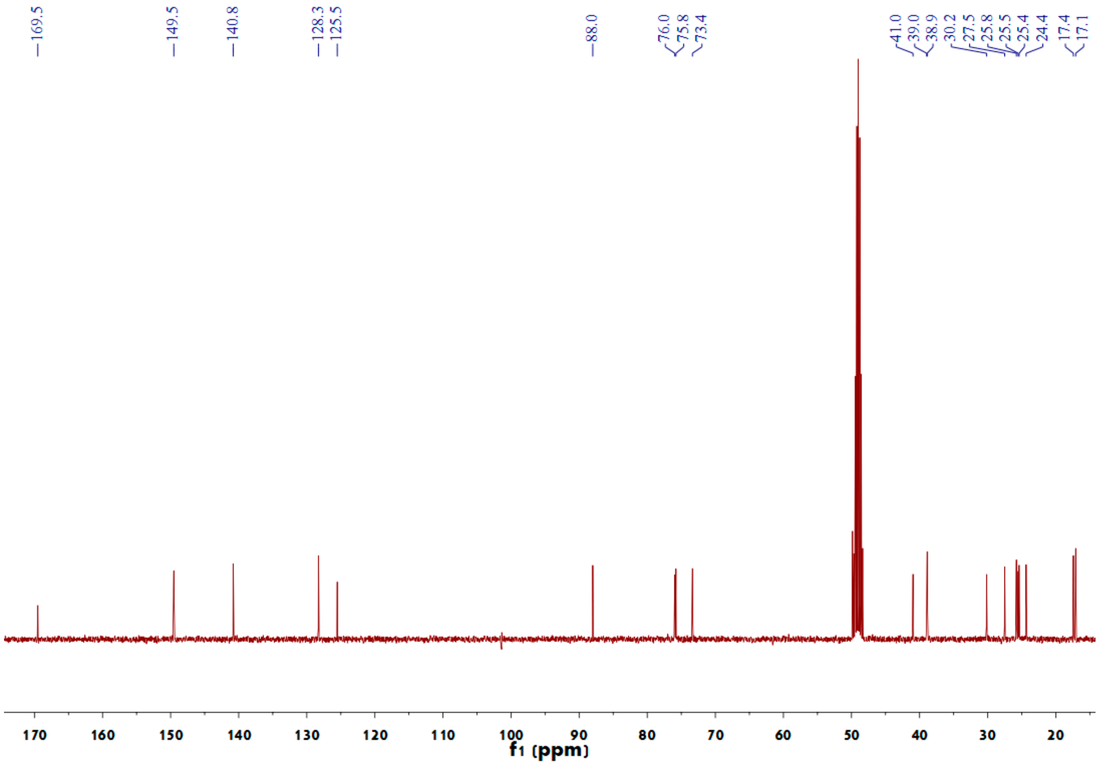

Figure S14. ^{13}C NMR (100 MHz, CD_3OD) spectrum of sarcophelegan C (**3**).

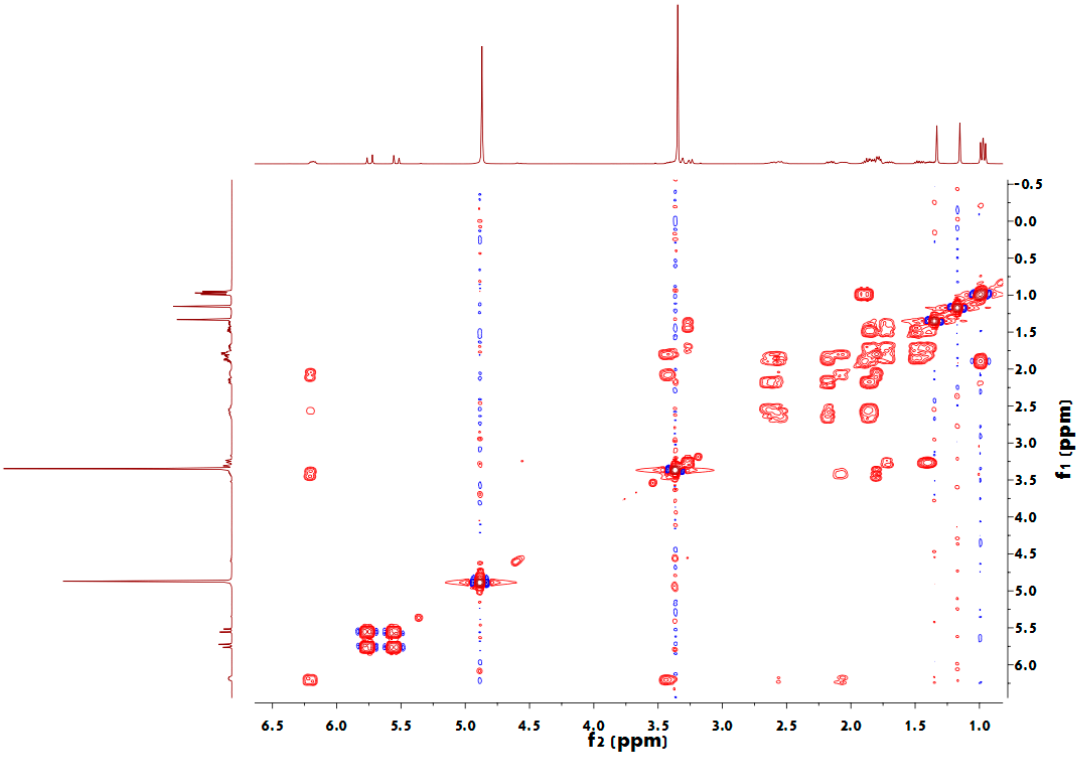

Figure S15. ^{1}H-^{1}H COSY spectrum of sarcophelegan C (**3**) in CD_3OD.

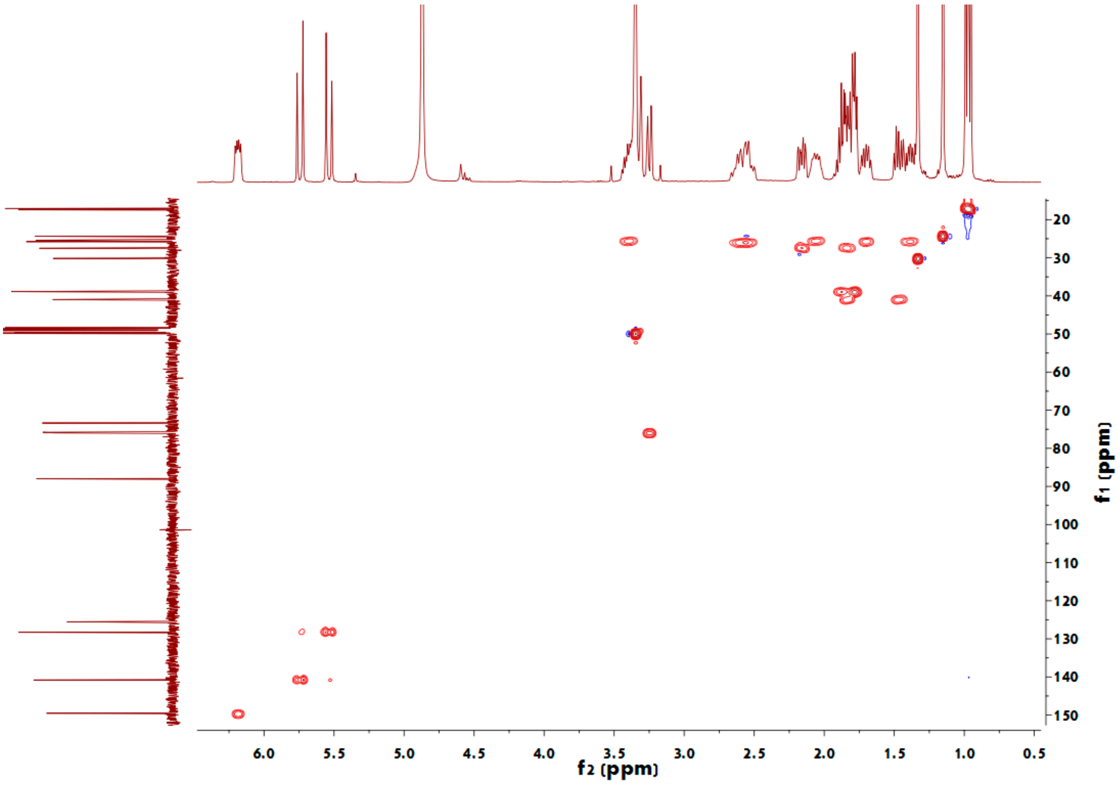

Figure S16. HSQC spectrum of sarcophelegan C (**3**) in CD_3OD.

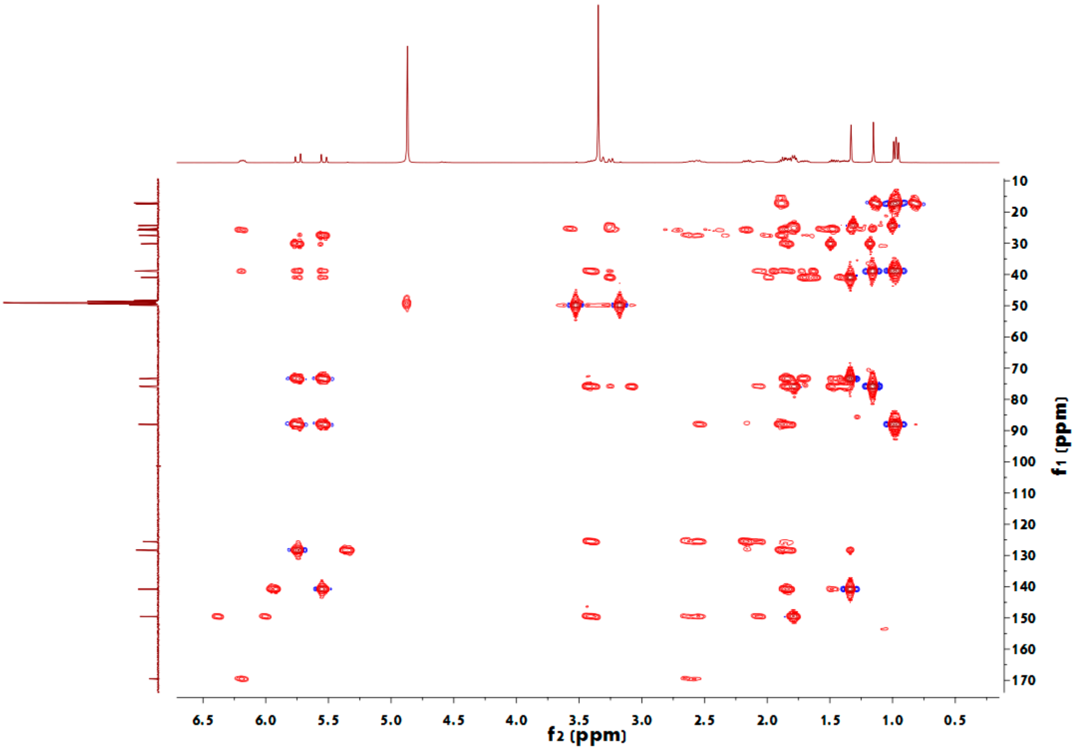

Figure S17. HMBC spectrum of sarcophelegan C (**3**) in CD_3OD.

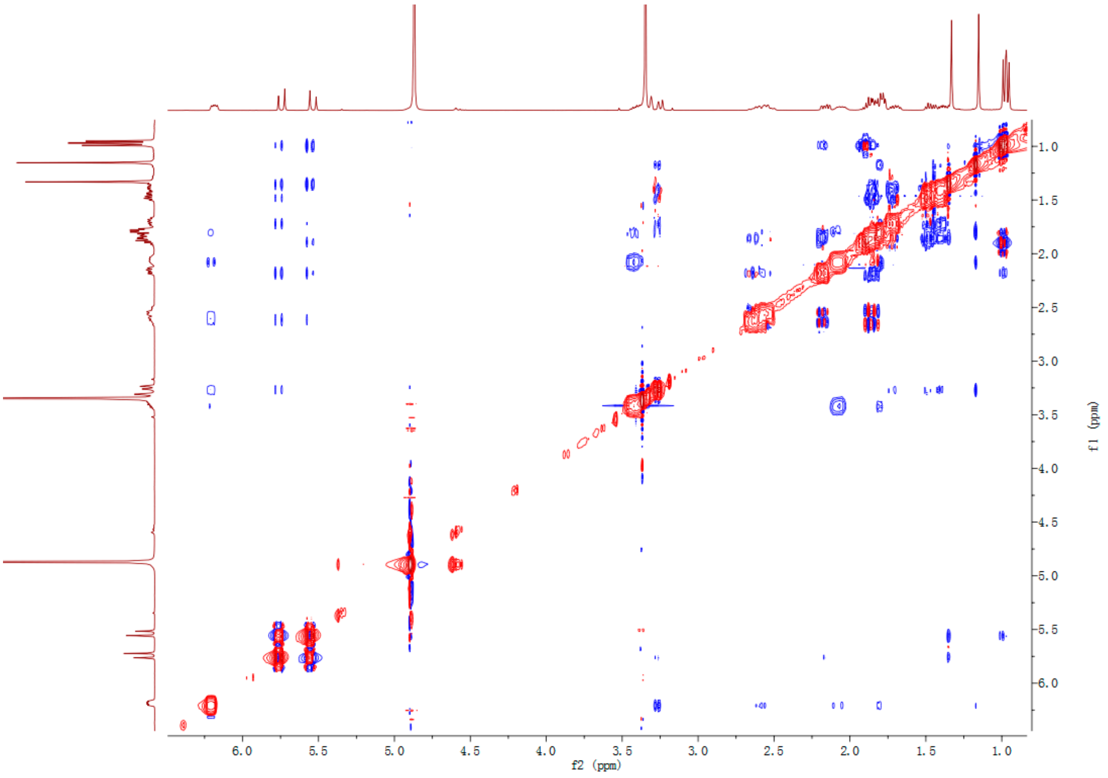

Figure S18. NOESY spectrum of sarcophelegan C (**3**) in CD_3OD.

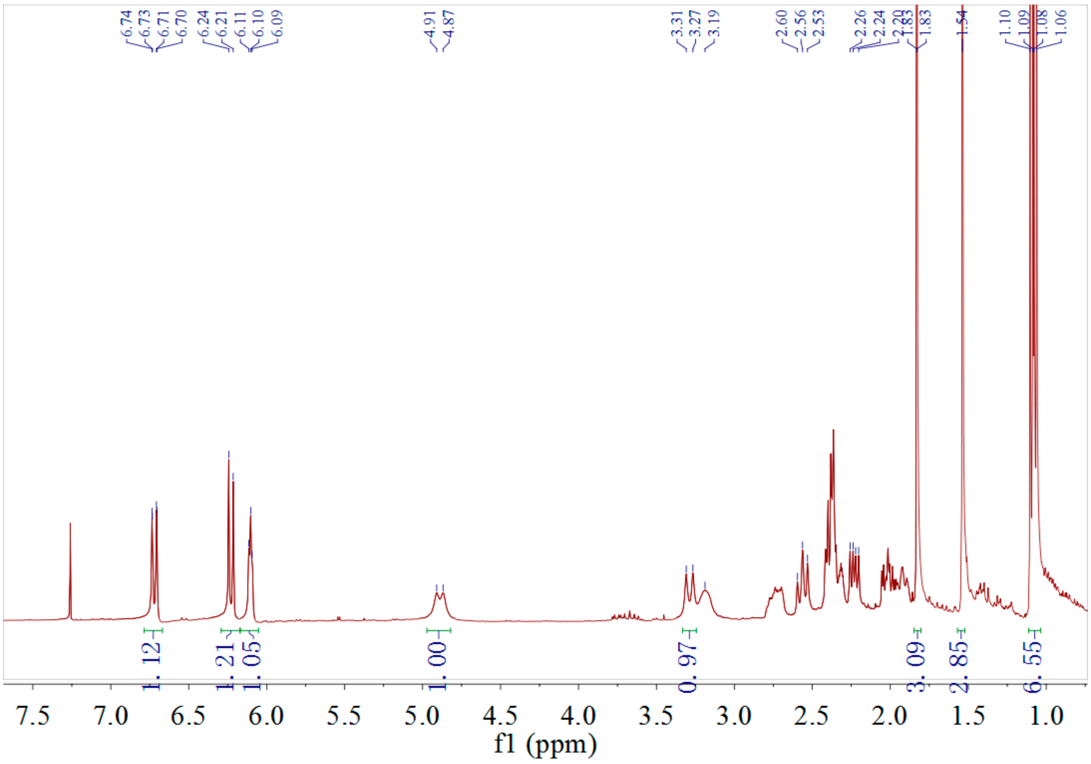

Figure S19. ^{1}H NMR (400 MHz, $CDCl_3$) spectrum of sarcophelegan D (**4**).

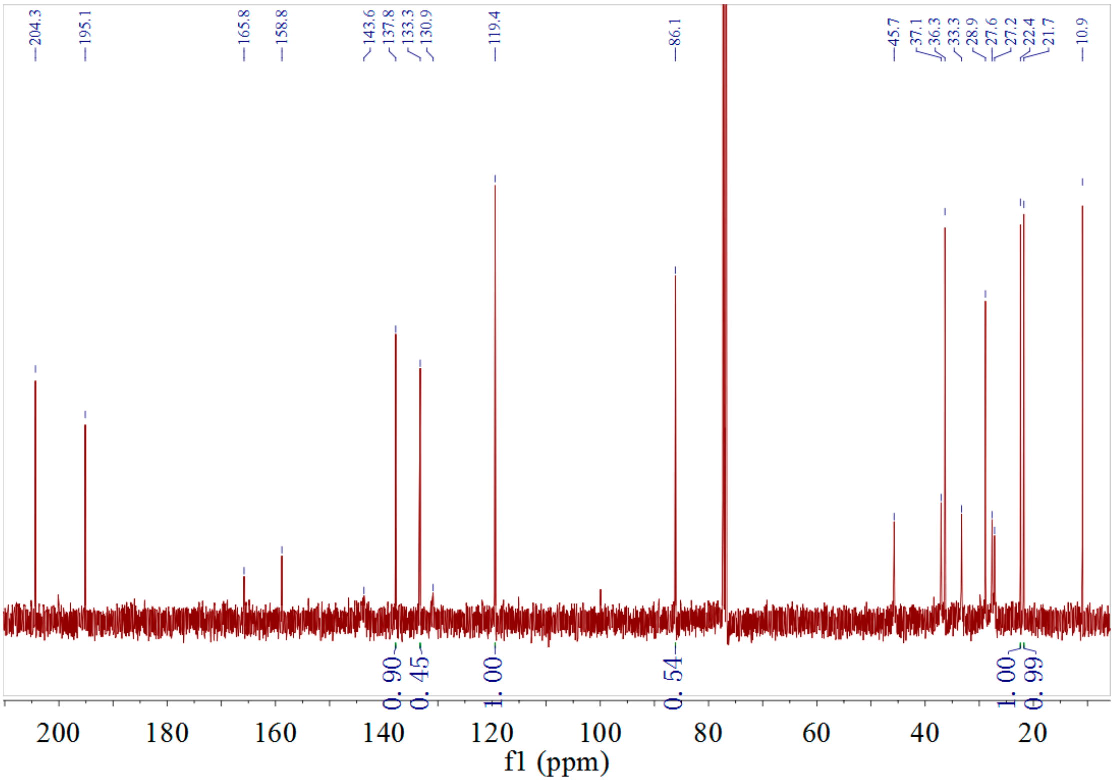

Figure S20. ^{13}C NMR (100 MHz, $CDCl_3$) spectrum of sarcophelegan D (**4**).

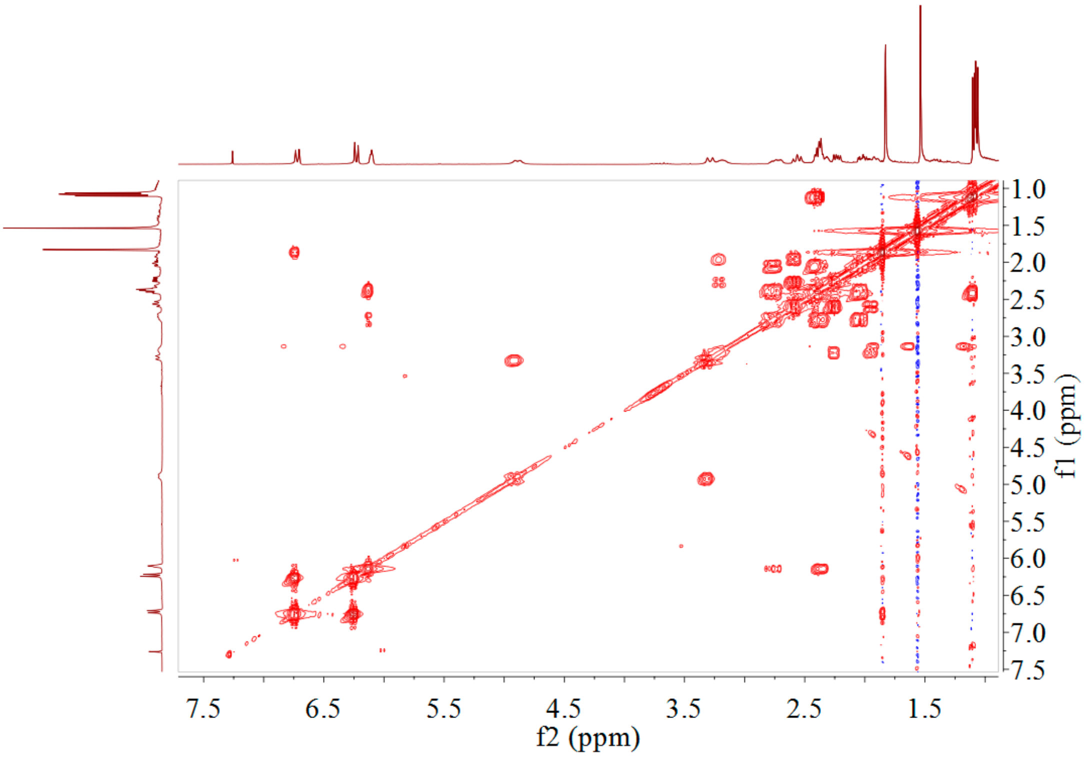

Figure S21. ^{1}H-^{1}H COSY spectrum of sarcophelegan D (**4**) in $CDCl_3$.

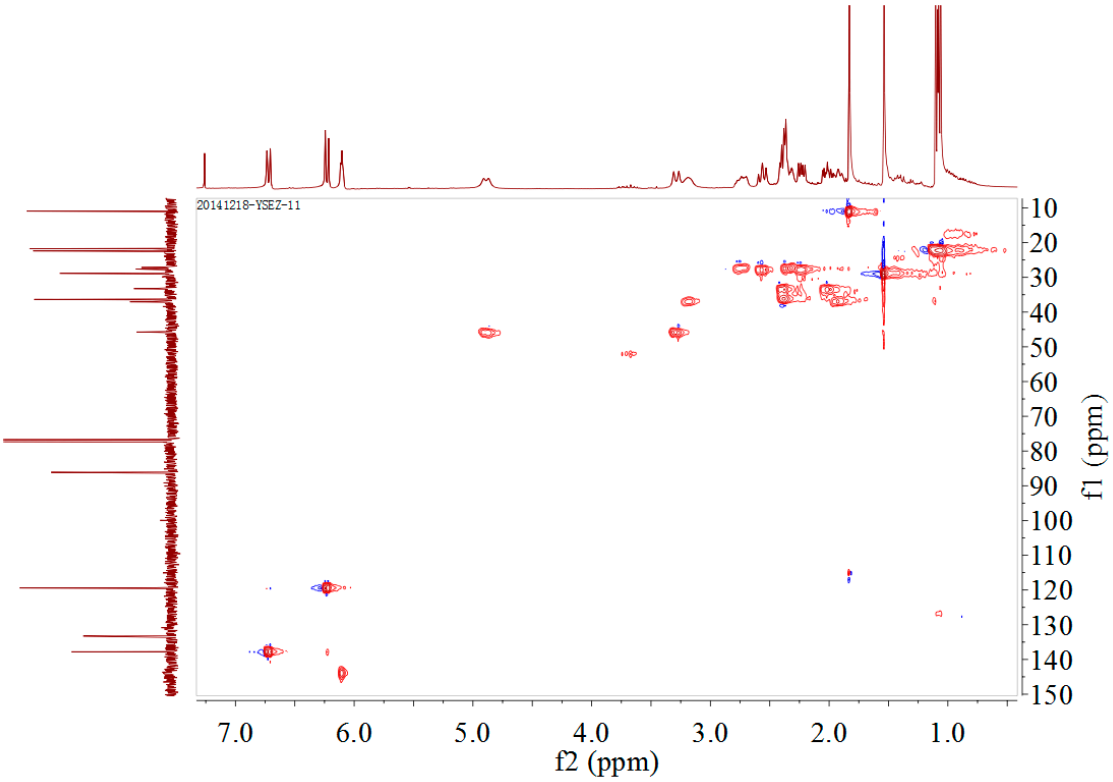

Figure S22. HSQC spectrum of sarcophelegan D (**4**) in $CDCl_3$.

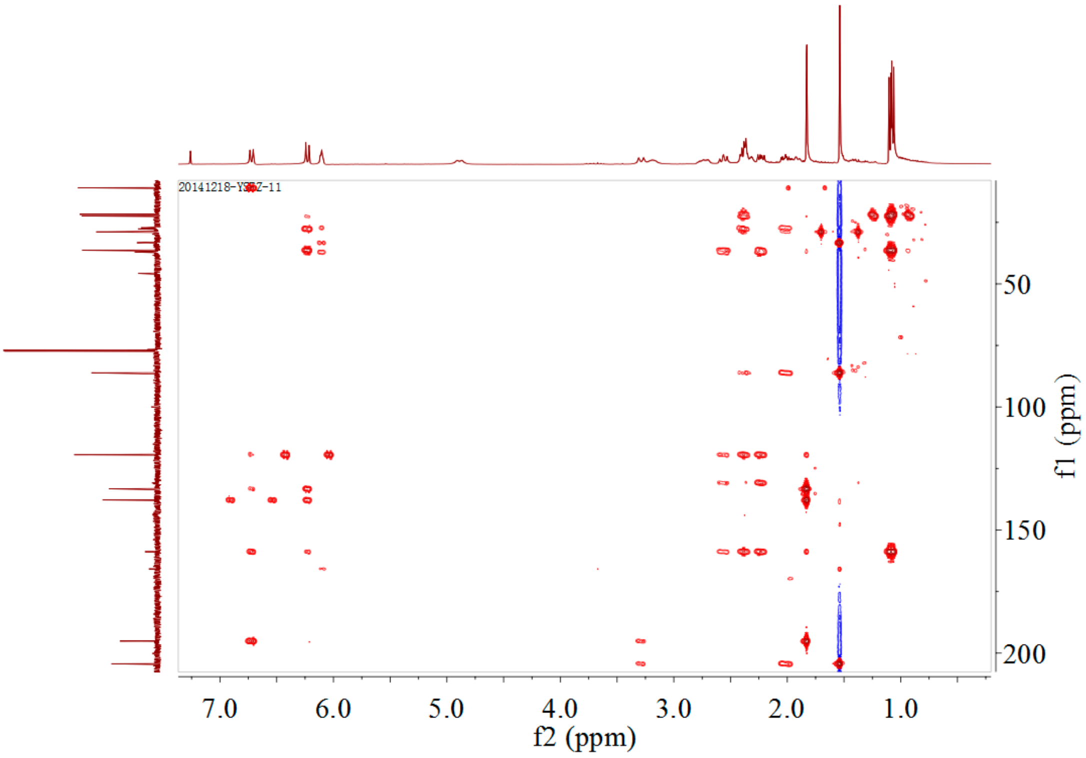

Figure S23. HMBC spectrum of sarcophelegan D (**4**) in $CDCl_3$.

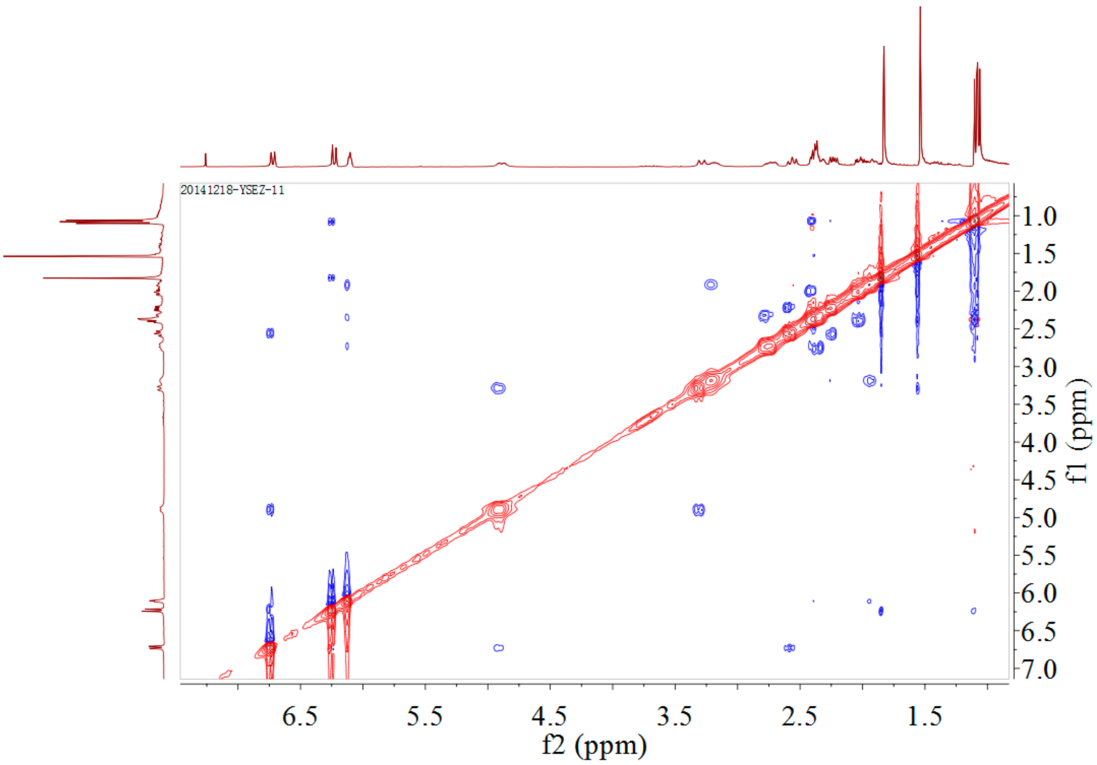

Figure S24. NOESY spectrum of sarcophelegan D (**4**) in $CDCl_3$.

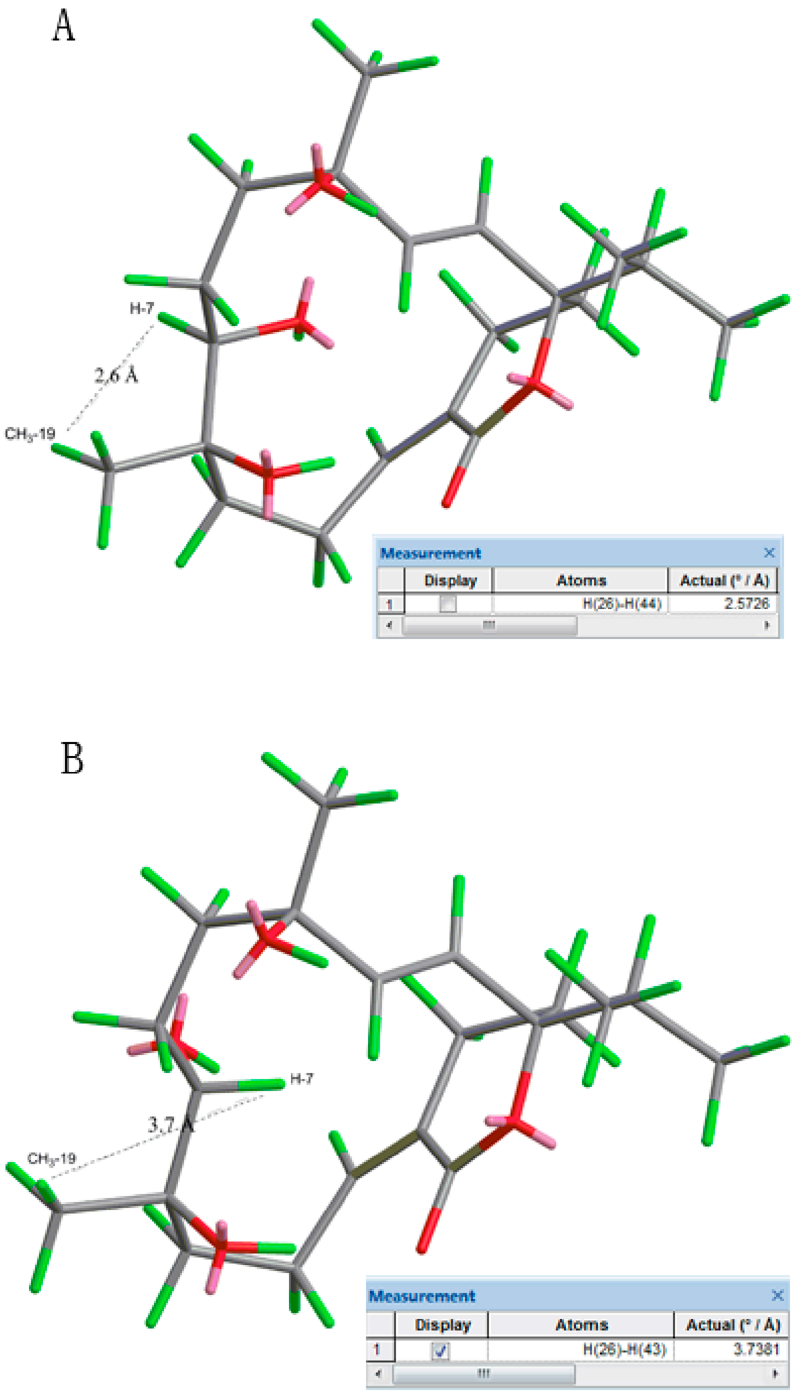

Figure S25. The Chem3D molecular modeling study of **3**. (**A**) repesents the distance between H-7 and CH_3-19 of 7β-OH isomer; (**B**) repesents the distance between H-7 and CH_3-19 of 7α-OH isomer.
